# Supplementary material for: Associations Between Physical Fitness and Brain Structure in Young Adulthood
Source: Front Psychol. 2020 Nov 17;11:608049. doi: 10.3389/fpsyg.2020.608049 (PMC7705380; doi:10.3389/fpsyg.2020.608049)
Supplement: Supplementary file 1 [file Data_Sheet_1.PDF]

Associations Between Physical Fitness and Brain Structure in Young Adulthood

Supplemental Material

John R. Best[1,2,3*], Elizabeth Dao[4], Ryan Churchill[2] and Theodore D. Cosco[1,2]
[1]Gerontology Research Centre, Simon Fraser University, Vancouver, British Columbia, Canada
[2]Department of Gerontology, Simon Fraser University, Vancouver, British Columbia, Canada
[3]Department of Psychiatry, University of British Columbia, Vancouver, British Columbia, Canada
[4]Department of Radiology, University of British Columbia, Vancouver, British Columbia, Canada

14 September 2020

Section 1: Comprehensive white matter fractional anisotropy results

The following tables summarize the associations of the three fitness measures (body mass index, submaximal cardiovascular endurance, and grip strength) with white matter fractional anisotropy. A multiple regression model was constructed for each brain ROI, including the three fitness measures, age, gender, annual income, educational attainment, race, gait speed and intra-cranial volume. Unstandardized beta coefficients and their standard errors are provided. Results are arranged from largest |z| statistic to the smallest |z| statistic.

Supplemental Table 1. Summary of effects of body mass index on white matter fractional anisotropy values.

White matter ROI	Effect	S.E.	Z stat
Cerebral__peduncle__L	-0.0081	0.0011	-7.7
Fornix__cres__L	-0.01	0.0016	-6.5
Fornix__cres__R	-0.0092	0.0016	-5.9
Cerebral__peduncle__R	-0.0063	0.0011	-5.8
Pontine__crossing__tract	-0.0074	0.0015	-5
Sagittal__stratum__L	-0.0072	0.0015	-4.9
Cingulum__hippocampus__R	-0.0094	0.002	-4.7
Retrolenticular__part__internal__capsule__R	-0.0062	0.0014	-4.4
Cingulum__hippocampus__L	-0.0082	0.0019	-4.3
Corticospinal__tract__L	-0.0073	0.0018	-4.1
Superior__frontooccipital__fasciculus__R	-0.0057	0.0015	-3.9
Inferior__cerebellar__peduncle__R	-0.0072	0.0018	-3.9
Superior__corona__radiata__R	-0.0045	0.0012	-3.9
Superior__longitudinal__fasciculus__R	-0.0045	0.0012	-3.8
Corticospinal__tract__R	-0.0069	0.0019	-3.7
Medial__lemniscus__R	-0.0056	0.0015	-3.6
Inferior__cerebellar__peduncle__L	-0.0063	0.0018	-3.5
Sagittal__stratum__R	-0.0047	0.0014	-3.3
Medial__lemniscus__L	-0.0048	0.0015	-3.3

*Correspondence: john[underscore]best@sfu.ca

White matter ROI	Effect	S.E.	Z stat
Posterior__limb__internal__capsule__R	-0.0038	0.0012	-3.2
Superior__cerebellar__peduncle__L	-0.0062	0.002	-3.1
Retrolenticular__part__internal__capsule__L	-0.0039	0.0013	-2.9
Superior__cerebellar__peduncle__R	-0.0058	0.002	-2.9
Uncinate__fasciculus__L	-0.0065	0.0023	-2.8
Posterior__corona__radiata__R	-0.0035	0.0012	-2.8
Middle__cerebellar__peduncle	-0.0033	0.0012	-2.8
Posterior__thalamic__radiation__R	-0.0034	0.0012	-2.8
External__capsule__L	-0.0025	0.001	-2.4
External__capsule__R	-0.0023	0.0011	-2.2
Anterior__limb__internal__capsule__R	-0.0027	0.0013	-2.1
Anterior__corona__radiata__R	-0.0028	0.0014	-2
Cingulum__cingulate__gyrus__L	0.0028	0.0017	1.6
Genu__corpus__callosum	-0.002	0.0013	-1.5
Posterior__thalamic__radiation__L	-0.0017	0.0013	-1.3
Tapetum__R	-0.0031	0.0024	-1.3
Superior__frontooccipital__fasciculus__L	-0.0024	0.002	-1.2
Anterior__corona__radiata__L	-0.0014	0.0013	-1.1
Posterior__corona__radiata__L	-0.0014	0.0013	-1.1
Superior__longitudinal__fasciculus__L	-0.0013	0.0012	-1
Cingulum__cingulate__gyrus__R	-0.0016	0.0016	-1
Uncinate__fasciculus__R	-0.0021	0.0021	-0.98
Splenium__corpus__callosum	-0.00091	0.001	-0.88
Posterior__limb__internal__capsule__L	-0.00099	0.0011	-0.88
Body__corpus__callosum	-0.00099	0.0012	-0.8
Anterior__limb__internal__capsule__L	-0.00083	0.0012	-0.7
Superior__corona__radiata__L	0.00057	0.0013	0.45
Fornix__column__and__body__fornix	0.00046	0.0037	0.12
Tapetum__L	-2e-04	0.0024	-0.083

Supplemental Table 2. Summary of effects of submaximal cardiovascular endurance on white matter fractional anisotropy values.

White matter ROI	Effect	S.E.	Z stat
Corticospinal__tract__R	0.0037	0.0018	2.1
Sagittal__stratum__L	-0.0028	0.0014	-2
Medial__lemniscus__L	0.0024	0.0014	1.8
Uncinate__fasciculus__R	-0.0035	0.002	-1.7
Fornix__column__and__body__fornix	0.0055	0.0036	1.5
Posterior__limb__internal__capsule__R	-0.0016	0.0011	-1.4
Fornix__cres__L	-0.002	0.0015	-1.3
Posterior__limb__internal__capsule__L	-0.0013	0.0011	-1.3
Medial__lemniscus__R	0.0017	0.0015	1.1
Corticospinal__tract__L	0.0018	0.0017	1.1
Uncinate__fasciculus__L	-0.0023	0.0022	-1.1
Superior__cerebellar__peduncle__R	0.0019	0.0019	0.99
Cingulum__hippocampus__L	-0.0017	0.0018	-0.97
Superior__frontooccipital__fasciculus__R	0.0013	0.0014	0.94
Posterior__thalamic__radiation__L	-0.001	0.0012	-0.87
External__capsule__R	0.00083	0.00099	0.83
Inferior__cerebellar__peduncle__L	0.0014	0.0017	0.82

White matter ROI	Effect	S.E.	Z stat
Retrolenticular__part__internal__capsule__L	-0.001	0.0012	-0.8
Posterior__thalamic__radiation__R	-0.00081	0.0011	-0.71
Splenium__corpus__callosum	-0.00065	0.00097	-0.67
Retrolenticular__part__internal__capsule__R	-0.00083	0.0013	-0.64
Superior__corona__radiata__L	0.00072	0.0012	0.61
Superior__cerebellar__peduncle__L	0.0011	0.0019	0.6
Genu__corpus__callosum	-0.00072	0.0012	-0.58
Posterior__corona__radiata__R	-0.00064	0.0011	-0.56
Superior__frontooccipital__fasciculus__L	0.001	0.0019	0.55
Anterior__limb__internal__capsule__L	6e-04	0.0011	0.54
Middle__cerebellar__peduncle	0.00058	0.0011	0.53
Tapetum__L	-0.0011	0.0022	-0.51
External__capsule__L	-0.00047	0.00097	-0.49
Posterior__corona__radiata__L	0.00058	0.0012	0.48
Cerebral__peduncle__R	-0.00048	0.001	-0.47
Anterior__limb__internal__capsule__R	5e-04	0.0012	0.43
Superior__longitudinal__fasciculus__R	-0.00045	0.0011	-0.41
Tapetum__R	-0.00091	0.0023	-0.4
Superior__longitudinal__fasciculus__L	0.00035	0.0012	0.3
Anterior__corona__radiata__R	0.00039	0.0013	0.3
Pontine__crossing__tract	0.00041	0.0014	0.3
Cingulum__cingulate__gyrus__R	-0.00031	0.0015	-0.21
Anterior__corona__radiata__L	-0.00023	0.0012	-0.19
Cerebral__peduncle__L	-0.00012	0.00098	-0.12
Superior__corona__radiata__R	-0.00012	0.0011	-0.11
Body__corpus__callosum	0.00011	0.0012	0.094
Cingulum__cingulate__gyrus__L	8.5e-05	0.0016	0.053
Sagittal__stratum__R	4.6e-05	0.0013	0.035
Inferior__cerebellar__peduncle__R	-4.5e-05	0.0017	-0.026
Fornix__cres__R	2.6e-05	0.0015	0.018
Cingulum__hippocampus__R	8.2e-06	0.0019	0.0044

Supplemental Table 3. Summary of effects of grip strength on white matter fractional anisotropy values.

White matter ROI	Effect	S.E.	Z stat
Cingulum__cingulate__gyrus__R	-0.0068	0.0022	-3.1
Superior__cerebellar__peduncle__L	-0.0087	0.0028	-3.1
Superior__cerebellar__peduncle__R	-0.0076	0.0028	-2.7
Middle__cerebellar__peduncle	-0.0041	0.0016	-2.6
Cingulum__hippocampus__L	-0.0063	0.0026	-2.4
Inferior__cerebellar__peduncle__L	-0.0057	0.0024	-2.3
Inferior__cerebellar__peduncle__R	-0.0056	0.0025	-2.2
Genu__corpus__callosum	-0.0039	0.0018	-2.2
Superior__longitudinal__fasciculus__R	-0.0033	0.0016	-2.1
Cingulum__cingulate__gyrus__L	-0.0048	0.0024	-2
Fornix__cres__L	-0.0043	0.0021	-2
Retrolenticular__part__internal__capsule__R	-0.0038	0.0019	-2
Posterior__thalamic__radiation__R	-0.0027	0.0017	-1.6
Fornix__cres__R	-0.0034	0.0021	-1.6
Superior__longitudinal__fasciculus__L	-0.0026	0.0017	-1.5
Pontine__crossing__tract	-0.0028	0.002	-1.4

White matter ROI	Effect	S.E.	Z stat
Fornix_column_and_body_fornix	-0.0069	0.0051	-1.4
Posterior_corona_radiata_R	-0.002	0.0017	-1.2
Uncinate_fasciculus_L	-0.0036	0.0031	-1.2
External_capsule_L	-0.0016	0.0014	-1.1
Body_corpus_callosum	-0.0018	0.0017	-1
Sagittal_stratum_R	-0.0019	0.0019	-1
Retrolenticular_part_internal_capsule_L	-0.0017	0.0018	-0.91
Tapetum_R	-0.003	0.0033	-0.91
External_capsule_R	-0.0013	0.0015	-0.91
Posterior_corona_radiata_L	-0.0015	0.0017	-0.85
Anterior_corona_radiata_L	0.0014	0.0018	0.77
Tapetum_L	0.0024	0.0032	0.73
Superior_corona_radiata_R	-0.0011	0.0016	-0.71
Corticospinal_tract_L	-0.0017	0.0024	-0.7
Anterior_limb_internal_capsule_L	-0.001	0.0016	-0.64
Corticospinal_tract_R	-0.0015	0.0026	-0.58
Superior_frontooccipital_fasciculus_R	-0.0012	0.002	-0.58
Medial_lemniscus_L	-0.0011	0.002	-0.56
Splenium_corpus_callosum	-0.00075	0.0014	-0.53
Cerebral_peduncle_R	-0.00076	0.0015	-0.51
Posterior_limb_internal_capsule_L	-0.00077	0.0015	-0.5
Cerebral_peduncle_L	-0.00063	0.0014	-0.44
Posterior_limb_internal_capsule_R	-0.00064	0.0016	-0.39
Medial_lemniscus_R	-8e-04	0.0021	-0.38
Anterior_corona_radiata_R	0.00067	0.0019	0.35
Anterior_limb_internal_capsule_R	-0.00051	0.0017	-0.29
Superior_frontooccipital_fasciculus_L	-0.00072	0.0027	-0.26
Posterior_thalamic_radiation_L	-0.00046	0.0018	-0.26
Uncinate_fasciculus_R	0.00074	0.0029	0.25
Cingulum_hippocampus_R	-0.00049	0.0028	-0.18
Superior_corona_radiata_L	1e-04	0.0017	0.06
Sagittal_stratum_L	1.1e-05	0.002	0.0055

Section 2: Comprehensive gray matter results

Gray matter cortical thickness The following tables summarize the associations of the three fitness measures (body mass index, submaximal cardiovascular endurance, and grip strength) with gray matter cortical thickness. A multiple regression model was constructed for each brain ROI, including the three fitness measures, age, gender, annual income, educational attainment, race, gait speed and intra-cranial volume.Unstandardized beta coefficients and their standard errors are provided. Results are arranged from largest |z| statistic to the smallest |z| statistic.

Supplemental Table 4. Summary of effects of body mass index on gray matter cortical thickness.

Gray matter thickness ROI	Effect	S.E.	Z stat
FS_R_Superiorparietal_Thck	0.022	0.0047	4.6
FS_L_Rostralanteriorcingulate_Thck	0.037	0.0093	4
FS_L_Temporalpole_Thck	-0.054	0.014	-3.9
FS_R_Rostralanteriorcingulate_Thck	0.039	0.01	3.8
FS_R_Lateralorbitofrontal_Thck	0.024	0.0062	3.8
FS_R_Inferiorparietal_Thck	0.018	0.0049	3.7

Gray matter thickness ROI	Effect	S.E.	Z stat
FS_L_Inferiorparietal_Thck	0.019	0.0053	3.6
FS_R_Entorhinal_Thck	-0.042	0.013	-3.3
FS_L_Superiorparietal_Thck	0.015	0.0049	3.1
FS_R_Temporalpole_Thck	-0.047	0.015	-3
FS_R_Medialorbitofrontal_Thck	0.02	0.0069	2.8
FS_L_Lingual_Thck	0.016	0.0055	2.8
FS_R_Supramarginal_Thck	0.015	0.0053	2.7
FS_L_Medialorbitofrontal_Thck	0.021	0.0079	2.6
FS_L_Lateralorbitofrontal_Thck	0.016	0.0061	2.6
FS_R_Lateraloccipital_Thck	0.013	0.0049	2.6
FS_L_Precuneus_Thck	0.014	0.0055	2.5
FS_R_Insula_Thck	0.017	0.0069	2.5
FS_L_Caudalanteriorcingulate_Thck	0.022	0.009	2.4
FS_L_Inferiortemporal_Thck	0.015	0.0063	2.4
FS_L_Rostralmiddlefrontal_Thck	0.013	0.0056	2.3
FS_L_Fusiform_Thck	0.014	0.0059	2.3
FS_L_Insula_Thck	0.015	0.0073	2.1
FS_L_Caudalmiddlefrontal_Thck	0.012	0.0059	2
FS_R_Parsorbitalis_Thck	0.015	0.0077	2
FS_L_Lateraloccipital_Thck	0.01	0.005	2
FS_L_Supramarginal_Thck	0.011	0.0055	2
FS_R_Inferiortemporal_Thck	0.012	0.0061	2
FS_L_Posteriorcingulate_Thck	0.013	0.0067	1.9
FS_R_Parstriangularis_Thck	0.011	0.006	1.8
FS_L_Parsopercularis_Thck	0.011	0.0061	1.8
FS_L_Entorhinal_Thck	-0.022	0.013	-1.8
FS_R_Postcentral_Thck	0.0082	0.0048	1.7
FS_L_Postcentral_Thck	0.0082	0.0048	1.7
FS_R_Middletemporal_Thck	0.01	0.006	1.7
FS_R_Cuneus_Thck	0.0092	0.0057	1.6
FS_R_Lingual_Thck	0.0084	0.0056	1.5
FS_L_Middletemporal_Thck	0.0095	0.0064	1.5
FS_R_Precuneus_Thck	0.0077	0.0054	1.4
FS_L_Bankssts_Thck	0.0099	0.007	1.4
FS_R_Rostralmiddlefrontal_Thck	0.0076	0.0054	1.4
FS_R_Isthmuscingulate_Thck	0.012	0.0089	1.4
FS_L_Parsorbitalis_Thck	0.011	0.0077	1.4
FS_L_Superiorfrontal_Thck	0.0084	0.0062	1.4
FS_L_Precentral_Thck	0.0074	0.0056	1.3
FS_R_Caudalanteriorcingulate_Thck	0.015	0.012	1.2
FS_R_Fusiform_Thck	0.0063	0.0055	1.1
FS_L_Isthmuscingulate_Thck	0.018	0.015	1.1
FS_R_Caudalmiddlefrontal_Thck	0.0063	0.0056	1.1
FS_L_Frontalpole_Thck	0.011	0.01	1.1
FS_L_Parahippocampal_Thck	0.014	0.013	1
FS_R_Parsopercularis_Thck	0.0061	0.006	1
FS_R_Bankssts_Thck	0.0061	0.0071	0.85
FS_R_Precentral_Thck	0.0041	0.0054	0.75
FS_L_Parstriangularis_Thck	0.0047	0.0063	0.75
FS_R_Superiorfrontal_Thck	0.0044	0.006	0.74
FS_R_Paracentral_Thck	0.0047	0.0063	0.74
FS_R_Frontalpole_Thck	0.0056	0.01	0.55

Gray matter thickness ROI	Effect	S.E.	Z stat
FS_L_Transversetemporal_Thck	-0.0043	0.0084	-0.51
FS_R_Pericalcarine_Thck	0.003	0.0062	0.48
FS_R_Posteriorcingulate_Thck	0.0036	0.0079	0.46
FS_R_Superiortemporal_Thck	0.0027	0.0061	0.45
FS_L_Superiortemporal_Thck	0.0028	0.0062	0.45
FS_R_Parahippocampal_Thck	0.0049	0.011	0.44
FS_L_Cuneus_Thck	0.0023	0.0056	0.41
FS_L_Paracentral_Thck	0.002	0.0065	0.31
FS_R_Transversetemporal_Thck	0.0021	0.0083	0.26
FS_L_Pericalcarine_Thck	0.0015	0.0059	0.26

Supplemental Table 5. Summary of effects of submaximal cardiovascular endurance on gray matter cortical thickness.

Gray matter thickness ROI	Effect	S.E.	Z stat
FS_R_Caudalanteriorcingulate_Thck	0.028	0.011	2.4
FS_R_Rostralanteriorcingulate_Thck	0.015	0.0096	1.5
FS_R_Superiortemporal_Thck	0.0083	0.0055	1.5
FS_R_Entorhinal_Thck	-0.017	0.012	-1.5
FS_L_Medialorbitofrontal_Thck	-0.0099	0.0074	-1.3
FS_R_Parsopercularis_Thck	0.0071	0.0055	1.3
FS_L_Paracentral_Thck	-0.0077	0.006	-1.3
FS_R_Bankssts_Thck	0.0082	0.0066	1.2
FS_L_Parsopercularis_Thck	0.0069	0.0056	1.2
FS_R_Temporalpole_Thck	0.017	0.014	1.2
FS_L_Entorhinal_Thck	-0.013	0.012	-1.1
FS_L_Caudalmiddlefrontal_Thck	-0.0057	0.0054	-1.1
FS_L_Insula_Thck	0.0068	0.0068	1
FS_R_Posteriorcingulate_Thck	0.0068	0.0073	0.93
FS_R_Lateralorbitofrontal_Thck	0.0053	0.0057	0.92
FS_L_Parahippocampal_Thck	0.011	0.012	0.9
FS_R_Cuneus_Thck	0.0046	0.0052	0.89
FS_L_Temporalpole_Thck	-0.011	0.013	-0.88
FS_L_Transversetemporal_Thck	-0.0067	0.0077	-0.86
FS_R_Postcentral_Thck	0.0036	0.0044	0.83
FS_L_Caudalanteriorcingulate_Thck	0.0067	0.0084	0.81
FS_L_Precuneus_Thck	-0.0039	0.005	-0.77
FS_L_Isthmuscingulate_Thck	-0.011	0.015	-0.76
FS_R_Parahippocampal_Thck	-0.0074	0.01	-0.73
FS_R_Superiorparietal_Thck	0.003	0.0043	0.7
FS_R_Pericalcarine_Thck	0.0038	0.0057	0.67
FS_R_Frontalpole_Thck	-0.0062	0.0094	-0.66
FS_L_Superiortemporal_Thck	-0.0038	0.0057	-0.66
FS_R_Insula_Thck	0.0041	0.0065	0.64
FS_R_Lateraloccipital_Thck	0.0028	0.0045	0.64
FS_L_Bankssts_Thck	-0.0039	0.0064	-0.61
FS_L_Superiorfrontal_Thck	-0.0032	0.0056	-0.57
FS_R_Precuneus_Thck	0.0025	0.0049	0.51
FS_R_Superiorfrontal_Thck	-0.0027	0.0054	-0.5
FS_R_Precentral_Thck	0.0024	0.0049	0.49
FS_L_Cuneus_Thck	-0.0024	0.0051	-0.46

Gray matter thickness ROI	Effect	S.E.	Z stat
FS_L_Inferiorparietal_Thck	0.0023	0.0049	0.46
FS_L_Lateraloccipital_Thck	-0.0021	0.0046	-0.45
FS_L_Precentral_Thck	0.0022	0.0051	0.43
FS_L_Posteriorcingulate_Thck	-0.0026	0.0062	-0.43
FS_L_Lingual_Thck	-0.0022	0.0051	-0.42
FS_R_Medialorbitofrontal_Thck	-0.0026	0.0063	-0.4
FS_L_Middletemporal_Thck	-0.0023	0.0058	-0.4
FS_R_Parsorbitalis_Thck	0.0028	0.0071	0.4
FS_L_Lateralorbitofrontal_Thck	0.002	0.0056	0.35
FS_L_Rostralanteriorcingulate_Thck	0.003	0.0086	0.35
FS_R_Paracentral_Thck	0.002	0.0058	0.34
FS_R_Middletemporal_Thck	0.0016	0.0055	0.28
FS_R_Inferiorparietal_Thck	0.0013	0.0045	0.28
FS_L_Postcentral_Thck	-0.0011	0.0044	-0.26
FS_L_Supramarginal_Thck	-0.0012	0.0051	-0.24
FS_R_Inferiortemporal_Thck	0.0012	0.0056	0.21
FS_R_Isthmuscingulate_Thck	0.0017	0.0083	0.2
FS_R_Fusiform_Thck	0.001	0.005	0.2
FS_R_Caudalmiddlefrontal_Thck	-0.001	0.0051	-0.2
FS_L_Parsorbitalis_Thck	-0.0014	0.0071	-0.19
FS_L_Parstriangularis_Thck	-0.001	0.0058	-0.18
FS_L_Inferiortemporal_Thck	-0.001	0.0059	-0.18
FS_R_Transversetemporal_Thck	0.0011	0.0076	0.15
FS_L_Rostralmiddlefrontal_Thck	0.00075	0.0051	0.15
FS_R_Supramarginal_Thck	0.00068	0.0049	0.14
FS_L_Fusiform_Thck	-0.00068	0.0055	-0.13
FS_R_Parstriangularis_Thck	0.00057	0.0055	0.1
FS_L_Frontalpole_Thck	-0.00079	0.0096	-0.083
FS_L_Superiorparietal_Thck	-0.00032	0.0045	-0.07
FS_R_Lingual_Thck	0.00028	0.0051	0.055
FS_R_Rostralmiddlefrontal_Thck	-0.00014	0.0049	-0.029
FS_L_Pericalcarine_Thck	0.00015	0.0055	0.027

Supplemental Table 6. Summary of effects of grip strength on gray matter cortical thickness.

Gray matter thickness ROI	Effect	S.E.	Z stat
FS_L_Superiortemporal_Thck	-0.019	0.0085	-2.2
FS_R_Temporalpole_Thck	-0.039	0.021	-1.9
FS_L_Paracentral_Thck	0.013	0.0088	1.5
FS_R_Superiortemporal_Thck	-0.012	0.0082	-1.5
FS_R_Superiorparietal_Thck	0.0088	0.0064	1.4
FS_R_Supramarginal_Thck	0.0096	0.0072	1.3
FS_L_Precuneus_Thck	0.0093	0.0074	1.3
FS_R_Caudalanteriorcingulate_Thck	-0.021	0.017	-1.2
FS_R_Rostralanteriorcingulate_Thck	-0.017	0.014	-1.2
FS_R_Medialorbitofrontal_Thck	0.01	0.0093	1.1
FS_R_Precuneus_Thck	0.0081	0.0073	1.1
FS_R_Caudalmiddlefrontal_Thck	-0.0081	0.0076	-1.1
FS_L_Middletemporal_Thck	-0.0091	0.0086	-1.1
FS_L_Superiorparietal_Thck	0.0069	0.0067	1
FS_L_Precentral_Thck	0.0076	0.0076	1

Gray matter thickness ROI	Effect	S.E.	Z stat
FS_L_Supramarginal_Thck	0.007	0.0075	0.93
FS_R_Lingual_Thck	-0.007	0.0076	-0.92
FS_R_Posteriorcingulate_Thck	-0.0098	0.011	-0.92
FS_R_Frontalpole_Thck	0.013	0.014	0.91
FS_L_Entorhinal_Thck	-0.016	0.017	-0.91
FS_L_Temporalpole_Thck	-0.017	0.019	-0.89
FS_R_Fusiform_Thck	-0.0066	0.0074	-0.88
FS_R_Lateraloccipital_Thck	0.0058	0.0066	0.87
FS_L_Bankssts_Thck	-0.0078	0.0095	-0.82
FS_R_Parsopercularis_Thck	-0.0063	0.0082	-0.78
FS_R_Middletemporal_Thck	-0.0062	0.0082	-0.76
FS_L_Parsopercularis_Thck	-0.0059	0.0082	-0.72
FS_R_Superiorfrontal_Thck	0.0056	0.0081	0.7
FS_L_Medialorbitofrontal_Thck	-0.0075	0.011	-0.7
FS_L_Parsorbitalis_Thck	-0.007	0.01	-0.67
FS_R_Transversetemporal_Thck	0.0069	0.011	0.62
FS_R_Isthmuscingulate_Thck	-0.0067	0.012	-0.55
FS_R_Inferiorparietal_Thck	0.0036	0.0066	0.55
FS_L_Parstriangularis_Thck	0.0046	0.0085	0.54
FS_R_Pericalcarine_Thck	0.0044	0.0083	0.53
FS_L_Superiorfrontal_Thck	0.0044	0.0084	0.52
FS_L_Frontalpole_Thck	-0.0069	0.014	-0.49
FS_L_Parahippocampal_Thck	0.0086	0.018	0.47
FS_L_Transversetemporal_Thck	-0.0053	0.011	-0.46
FS_L_Postcentral_Thck	0.003	0.0065	0.46
FS_L_Posteriorcingulate_Thck	-0.0042	0.0091	-0.46
FS_L_Caudalanteriorcingulate_Thck	0.0055	0.012	0.45
FS_R_Lateralorbitofrontal_Thck	-0.0037	0.0084	-0.44
FS_R_Paracentral_Thck	0.0036	0.0086	0.42
FS_L_Inferiortemporal_Thck	-0.0035	0.0086	-0.41
FS_L_Cuneus_Thck	0.003	0.0076	0.4
FS_L_Pericalcarine_Thck	0.0032	0.008	0.4
FS_R_Inferiortemporal_Thck	-0.0032	0.0083	-0.38
FS_L_Rostralmiddlefrontal_Thck	0.0028	0.0075	0.37
FS_L_Lingual_Thck	-0.0026	0.0075	-0.35
FS_R_Entorhinal_Thck	0.0058	0.017	0.34
FS_R_Precentral_Thck	0.0025	0.0073	0.34
FS_R_Rostralmiddlefrontal_Thck	0.0023	0.0073	0.32
FS_L_Lateralorbitofrontal_Thck	-0.0025	0.0083	-0.3
FS_R_Parahippocampal_Thck	0.0041	0.015	0.28
FS_R_Cuneus_Thck	-0.002	0.0077	-0.26
FS_L_Lateraloccipital_Thck	0.0015	0.0068	0.22
FS_L_Fusiform_Thck	0.00083	0.008	0.1
FS_R_Postcentral_Thck	0.00065	0.0065	0.1
FS_R_Insula_Thck	0.00091	0.0094	0.096
FS_L_Caudalmiddlefrontal_Thck	0.00061	0.008	0.076
FS_R_Parstriangularis_Thck	-0.00059	0.0081	-0.073
FS_L_Inferiorparietal_Thck	0.00046	0.0073	0.064
FS_R_Parsorbitalis_Thck	0.00057	0.01	0.055
FS_L_Insula_Thck	-0.00052	0.0099	-0.053
FS_R_Bankssts_Thck	0.00041	0.0097	0.042
FS_L_Isthmuscingulate_Thck	-0.00053	0.021	-0.025

Gray matter thickness ROI	Effect	S.E.	Z stat
FS_L_Rostralanteriorcingulate_Thck	-9.2e-05	0.013	-0.0073

Gray matter cortical area The following tables summarize the associations of the three fitness measures (body mass index, submaximal cardiovascular endurance, and grip strength) with gray matter cortical area. A multiple regression model was constructed for each brain ROI, including the three fitness measures, age, gender, annual income, educational attainment, race, gait speed and intra-cranial volume. Unstandardized beta coefficients and their standard errors are provided. Results are arranged from largest |z| statistic to the smallest |z| statistic.

Supplemental Table 7. Summary of effects of body mass index on gray matter cortical area.

Gray matter area ROI	Effect	S.E.	Z stat
FS_L_Entorhinal_Area	-11	3.6	-3
FS_L_Cuneus_Area	-21	8.8	-2.5
FS_L_Insula_Area	-25	11	-2.2
FS_R_Supramarginal_Area	-45	23	-2
FS_L_Pericalcarine_Area	-20	10	-1.9
FS_L_Fusiform_Area	-35	18	-1.9
FS_R_Entorhinal_Area	-7.4	4	-1.9
FS_L_Lingual_Area	-33	19	-1.8
FS_L_Inferiortemporal_Area	-35	20	-1.8
FS_L_Postcentral_Area	-33	19	-1.8
FS_R_Middletemporal_Area	-29	17	-1.7
FS_R_Lingual_Area	-30	18	-1.7
FS_R_Pericalcarine_Area	-18	11	-1.6
FS_L_Temporalpole_Area	-4.5	2.8	-1.6
FS_L_Parahippocampal_Area	-8.1	5.2	-1.6
FS_R_Temporalpole_Area	-4.4	2.8	-1.6
FS_R_Cuneus_Area	-16	10	-1.5
FS_R_Isthmuscingulate_Area	11	7.5	1.5
FS_L_Middletemporal_Area	-23	16	-1.5
FS_R_Superiorparietal_Area	-37	26	-1.4
FS_L_Lateraloccipital_Area	-34	24	-1.4
FS_R_Parahippocampal_Area	-5.9	4.5	-1.3
FS_L_Superiortemporal_Area	-22	17	-1.3
FS_L_Medialorbitofrontal_Area	16	13	1.3
FS_L_Paracentral_Area	10	8.4	1.2
FS_R_Fusiform_Area	-19	18	-1.1
FS_R_Parstriangularis_Area	-12	12	-1
FS_R_Posteriorcingulate_Area	-9.6	9.6	-0.99
FS_L_Parsopercularis_Area	-11	13	-0.9
FS_R_Inferiorparietal_Area	-26	29	-0.89
FS_R_Lateralorbitofrontal_Area	-11	12	-0.88
FS_R_Transversetemporal_Area	-2.2	2.5	-0.88
FS_L_Parsorbitalis_Area	3.1	3.7	0.86
FS_L_Lateralorbitofrontal_Area	-8.6	11	-0.77
FS_R_Caudalmiddlefrontal_Area	13	17	0.75
FS_R_Inferiortemporal_Area	-13	19	-0.71
FS_R_Paracentral_Area	6.3	10	0.63
FS_L_Inferiorparietal_Area	-16	26	-0.62
FS_R_Precentral_Area	13	21	0.61

Gray matter area ROI	Effect	S.E.	Z stat
FS_R_Postcentral_Area	-11	19	-0.58
FS_R_Insula_Area	-6	11	-0.53
FS_R_Frontalpole_Area	1.1	2.1	0.52
FS_L_Caudalanteriorcingulate_Area	-3.6	7	-0.52
FS_R_Superiortemporal_Area	-7.8	15	-0.5
FS_L_Caudalmiddlefrontal_Area	-9	18	-0.5
FS_R_Bankssts_Area	3.1	6.4	0.49
FS_R_Parsorbitalis_Area	-2.2	4.5	-0.48
FS_L_Rostralmiddlefrontal_Area	13	29	0.43
FS_L_Parstriangularis_Area	-3.8	9.2	-0.42
FS_R_Superiorfrontal_Area	12	31	0.4
FS_R_Medialorbitofrontal_Area	-3.4	9.1	-0.38
FS_L_Isthmuscingulate_Area	-6.6	19	-0.35
FS_R_Precuneus_Area	-6.3	21	-0.3
FS_L_Transversetemporal_Area	-1	3.5	-0.29
FS_R_Caudalanteriorcingulate_Area	-2.6	10	-0.25
FS_L_Frontalpole_Area	0.3	1.7	0.18
FS_L_Bankssts_Area	-1.2	7.7	-0.16
FS_R_Rostralmiddlefrontal_Area	-4.2	31	-0.14
FS_L_Superiorfrontal_Area	-4	32	-0.13
FS_L_Precuneus_Area	-1.3	18	-0.073
FS_R_Lateraloccipital_Area	-1.8	26	-0.069
FS_L_Precentral_Area	-1.1	21	-0.054
FS_L_Supramarginal_Area	0.78	22	0.035
FS_R_Rostralanteriorcingulate_Area	-0.13	6.3	-0.021
FS_R_Parsopercularis_Area	-0.22	11	-0.02
FS_L_Posteriorcingulate_Area	0.13	8.5	0.015
FS_L_Rostralanteriorcingulate_Area	-0.095	6.9	-0.014
FS_L_Superiorparietal_Area	-5.1e-05	27	-1.9e-06

Supplemental Table 8. Summary of effects of submaximal cardiovascular endurance on gray matter cortical area.

Gray matter area ROI	Effect	S.E.	Z stat
FS_R_Inferiorparietal_Area	75	27	2.8
FS_R_Superiorfrontal_Area	70	29	2.5
FS_L_Entorhinal_Area	6.4	3.4	1.9
FS_L_Isthmuscingulate_Area	33	18	1.9
FS_R_Bankssts_Area	10	6	1.7
FS_L_Precentral_Area	30	19	1.6
FS_L_Fusiform_Area	25	17	1.5
FS_L_Transversetemporal_Area	4.6	3.2	1.4
FS_R_Middletemporal_Area	21	15	1.4
FS_R_Lateralorbitofrontal_Area	15	11	1.3
FS_L_Rostralanteriorcingulate_Area	8.3	6.4	1.3
FS_L_Inferiorparietal_Area	32	24	1.3
FS_R_Lingual_Area	21	16	1.3
FS_L_Lateralorbitofrontal_Area	13	10	1.2
FS_R_Paracentral_Area	11	9.2	1.2
FS_L_Supramarginal_Area	24	21	1.2
FS_R_Transversetemporal_Area	-2.7	2.3	-1.1

Gray matter area ROI	Effect	S.E.	Z stat
FS_L_Superiorparietal_Area	27	24	1.1
FS_L_Inferiortemporal_Area	20	18	1.1
FS_R_Parsopercularis_Area	10	10	0.99
FS_R_Precentral_Area	19	19	0.97
FS_L_Caudalmiddlefrontal_Area	15	17	0.89
FS_R_Medialorbitofrontal_Area	7.2	8.4	0.86
FS_R_Caudalmiddlefrontal_Area	13	16	0.83
FS_L_Superiorfrontal_Area	24	29	0.82
FS_R_Lateraloccipital_Area	20	24	0.82
FS_R_Frontalpole_Area	-1.6	2	-0.82
FS_R_Insula_Area	8.1	10	0.78
FS_L_Frontalpole_Area	1.2	1.6	0.78
FS_R_Superiortemporal_Area	-11	14	-0.75
FS_L_Medialorbitofrontal_Area	8.8	12	0.74
FS_R_Cuneus_Area	-6.9	9.4	-0.73
FS_R_Parsorbitalis_Area	3	4.2	0.71
FS_L_Paracentral_Area	-5.5	7.8	-0.71
FS_R_Rostralanteriorcingulate_Area	-4.2	5.9	-0.71
FS_L_Parstriangularis_Area	5.7	8.5	0.67
FS_L_Posteriorcingulate_Area	5	7.8	0.64
FS_R_Inferiortemporal_Area	11	17	0.62
FS_R_Entorhinal_Area	-2.3	3.7	-0.61
FS_L_Precuneus_Area	10	17	0.6
FS_R_Parahippocampal_Area	2.5	4.1	0.6
FS_L_Middletemporal_Area	8.6	14	0.59
FS_L_Lateraloccipital_Area	13	22	0.59
FS_L_Superiortemporal_Area	-7.8	16	-0.5
FS_L_Insula_Area	-5	10	-0.49
FS_R_Superiorparietal_Area	-11	24	-0.47
FS_R_Isthmuscingulate_Area	3.2	6.9	0.46
FS_L_Postcentral_Area	7.7	17	0.45
FS_R_Caudalanteriorcingulate_Area	-4.3	9.8	-0.44
FS_L_Temporalpole_Area	1.1	2.6	0.43
FS_L_Rostralmiddlefrontal_Area	11	27	0.4
FS_L_Parahippocampal_Area	1.6	4.8	0.34
FS_L_Cuneus_Area	-2.4	8	-0.3
FS_L_Parsopercularis_Area	3.2	12	0.27
FS_L_Bankssts_Area	-1.8	7.2	-0.25
FS_R_Temporalpole_Area	0.61	2.6	0.23
FS_L_Lingual_Area	-3.9	17	-0.23
FS_L_Parsorbitalis_Area	-0.76	3.4	-0.23
FS_R_Rostralmiddlefrontal_Area	6	28	0.21
FS_R_Fusiform_Area	2.9	16	0.18
FS_R_Precuneus_Area	-2.9	19	-0.15
FS_R_Pericalcarine_Area	1.5	10	0.15
FS_R_Postcentral_Area	-2.4	18	-0.14
FS_R_Supramarginal_Area	-2.8	21	-0.13
FS_R_Posteriorcingulate_Area	-0.83	9	-0.093
FS_R_Parstriangularis_Area	0.81	11	0.075
FS_L_Caudalanteriorcingulate_Area	-0.15	6.5	-0.024
FS_L_Pericalcarine_Area	-0.14	9.5	-0.015

Supplemental Table 9. Summary of effects of grip strength on gray matter cortical area.

Gray matter area ROI	Effect	S.E.	Z stat
FS_L_Cuneus_Area	34	12	2.9
FS_R_Inferiorparietal_Area	108	40	2.7
FS_R_Posteriorcingulate_Area	33	13	2.5
FS_L_Entorhinal_Area	11	4.9	2.2
FS_L_Inferiorparietal_Area	71	36	2
FS_L_Fusiform_Area	44	25	1.8
FS_L_Pericalcarine_Area	25	14	1.8
FS_R_Cuneus_Area	24	14	1.7
FS_R_Parsorbitalis_Area	11	6.2	1.7
FS_R_Fusiform_Area	35	24	1.4
FS_R_Superiortemporal_Area	30	21	1.4
FS_L_Posteriorcingulate_Area	16	12	1.4
FS_L_Parsopercularis_Area	24	17	1.4
FS_R_Lateralorbitofrontal_Area	23	17	1.4
FS_R_Pericalcarine_Area	21	15	1.4
FS_L_Parstriangularis_Area	17	13	1.3
FS_R_Caudalanteriorcingulate_Area	18	14	1.2
FS_L_Middletemporal_Area	26	21	1.2
FS_R_Medialorbitofrontal_Area	13	12	1.1
FS_R_Lateraloccipital_Area	-36	35	-1
FS_R_Rostralanteriorcingulate_Area	-8.1	8.6	-0.94
FS_R_Lingual_Area	22	24	0.92
FS_L_Insula_Area	13	15	0.88
FS_R_Temporalpole_Area	-3.3	3.9	-0.85
FS_L_Lateralorbitofrontal_Area	13	15	0.84
FS_R_Parahippocampal_Area	4.8	6.1	0.8
FS_R_Middletemporal_Area	18	23	0.78
FS_L_Frontalpole_Area	1.7	2.3	0.77
FS_R_Postcentral_Area	-20	26	-0.76
FS_R_Parsopercularis_Area	-11	15	-0.75
FS_L_Medialorbitofrontal_Area	-13	17	-0.75
FS_R_Entorhinal_Area	4	5.4	0.74
FS_R_Precuneus_Area	21	28	0.73
FS_R_Precentral_Area	-20	29	-0.72
FS_L_Caudalanteriorcingulate_Area	6.6	9.4	0.7
FS_L_Supramarginal_Area	21	30	0.69
FS_L_Temporalpole_Area	-2.6	3.8	-0.67
FS_R_Supramarginal_Area	21	31	0.67
FS_L_Rostralmiddlefrontal_Area	25	40	0.63
FS_R_Insula_Area	-9.3	15	-0.61
FS_R_Superiorfrontal_Area	26	42	0.61
FS_L_Superiorfrontal_Area	-24	43	-0.55
FS_L_Superiorparietal_Area	18	36	0.49
FS_R_Caudalmiddlefrontal_Area	11	23	0.47
FS_R_Parstriangularis_Area	7.4	16	0.47
FS_R_Paracentral_Area	-6.3	14	-0.46
FS_L_Transversetemporal_Area	2	4.7	0.43
FS_L_Precuneus_Area	10	25	0.42
FS_L_Postcentral_Area	-11	25	-0.41
FS_L_Inferiortemporal_Area	11	27	0.41
FS_L_Superiortemporal_Area	8.7	23	0.38

Gray matter area ROI	Effect	S.E.	Z stat
FS_R_Isthmuscingulate_Area	3.8	10	0.38
FS_L_Lateraloccipital_Area	12	33	0.35
FS_R_Frontalpole_Area	-0.74	2.9	-0.25
FS_R_Superiorparietal_Area	8.7	35	0.25
FS_L_Caudalmiddlefrontal_Area	-5.9	25	-0.24
FS_L_Parsorbitalis_Area	-1.1	5	-0.22
FS_R_Rostralmiddlefrontal_Area	-8.9	41	-0.22
FS_L_Paracentral_Area	-2.3	11	-0.21
FS_R_Bankssts_Area	1.6	8.7	0.19
FS_L_Lingual_Area	4.7	25	0.19
FS_L_Precentral_Area	-3.5	28	-0.12
FS_R_Transversetemporal_Area	-0.37	3.4	-0.11
FS_R_Inferiortemporal_Area	2.7	26	0.1
FS_L_Isthmuscingulate_Area	-2.5	26	-0.097
FS_L_Rostralanteriorcingulate_Area	-0.75	9.4	-0.08
FS_L_Bankssts_Area	0.46	10	0.044
FS_L_Parahippocampal_Area	-0.25	7.1	-0.035

Gray matter subcortical volume The following tables summarize the associations of the three fitness measures (body mass index, submaximal cardiovascular endurance, and grip strength) with gray matter subcortical volume A multiple regression model was constructed for each brain ROI, including the three fitness measures, age, gender, annual income, educational attainment, race, gait speed and intra-cranial volume. Unstandardized beta coefficients and their standard errors are provided. Results are arranged from largest |z| statistic to the smallest |z| statistic.

Supplemental Table 10. Summary of effects of body mass index on gray matter subcortical volume.

Gray matter volume ROI	Effect	S.E.	Z stat
FS_R_Caudate_Vol	36	19	1.9
FS_L_Caudate_Vol	28	18	1.5
FS_L_AccumbensArea_Vol	-6.2	4.1	-1.5
FS_R_AccumbensArea_Vol	-6.3	4.3	-1.4
FS_R_Hippo_Vol	-16	17	-0.99
FS_R_ThalamusProper_Vol	-25	29	-0.86
FS_L_Amygdala_Vol	6	7.4	0.82
FS_L_ThalamusProper_Vol	-23	34	-0.67
FS_R_Pallidum_Vol	-4.3	8.5	-0.51
FS_R_Putamen_Vol	-11	24	-0.44
FS_L_Hippo_Vol	-8.2	20	-0.42
FS_L_Pallidum_Vol	-4.1	11	-0.38
FS_R_Amygdala_Vol	1.4	8.3	0.17
FS_L_Putamen_Vol	3.5	31	0.11

Supplemental Table 11. Summary of effects of submaximal cardiovascular endurance on gray matter subcortical volume.

Gray matter volume ROI	Effect	S.E.	Z stat
FS_R_Putamen_Vol	30	22	1.4
FS_R_Pallidum_Vol	10	7.8	1.4
FS_L_Pallidum_Vol	13	9.9	1.3

Gray matter volume ROI	Effect	S.E.	Z stat
FS_R_Hippo_Vol	15	15	0.99
FS_R_AccumbensArea_Vol	3.9	4	0.98
FS_L_AccumbensArea_Vol	-3.4	3.8	-0.91
FS_L_Putamen_Vol	24	28	0.85
FS_L_Amygdala_Vol	-4.1	6.8	-0.61
FS_R_Caudate_Vol	6.1	17	0.36
FS_R_Amygdala_Vol	-2.1	7.6	-0.27
FS_R_ThalamusProper_Vol	6.3	26	0.24
FS_L_Hippo_Vol	3.9	18	0.22
FS_L_ThalamusProper_Vol	6.5	32	0.21
FS_L_Caudate_Vol	-0.12	17	-0.0072

Supplemental Table 12. Summary of effects of grip strength on gray matter subcortical volume.

Gray matter volume ROI	Effect	S.E.	Z stat
FS_R_Caudate_Vol	-53	25	-2.1
FS_L_Pallidum_Vol	-18	15	-1.2
FS_L_Caudate_Vol	-29	25	-1.2
FS_R_Amygdala_Vol	-11	11	-0.98
FS_R_Pallidum_Vol	-11	11	-0.96
FS_R_Putamen_Vol	-30	32	-0.92
FS_L_ThalamusProper_Vol	39	47	0.84
FS_R_AccumbensArea_Vol	3.8	5.9	0.64
FS_L_AccumbensArea_Vol	3.1	5.6	0.56
FS_L_Amygdala_Vol	-4.8	10	-0.48
FS_R_Hippo_Vol	-8.1	23	-0.36
FS_L_Putamen_Vol	-13	42	-0.32
FS_L_Hippo_Vol	-8	27	-0.3
FS_R_ThalamusProper_Vol	-1.7	39	-0.042

Section 3: Behavioral genetic model comparisons

In the first set of model comparisons, we compare a saturated model (labelled ‘SibSaturated’) in which factor loadings are freely estimated for each sibling in a pair and across pair type (monozygotic, dizygotic, or full-sibling, non-twins) versus a model constraining mean scores or variances to be equal across siblings within a pair (labelled ‘eqMeSibs’ and ‘eqThSibs’, respectively), or further, constraining these values to be equal across the three sibling pair types (labelled ‘eqMeZyg’ and ‘eqThZyg’, respectively).

A separate set of output is provided for each FA and cortical thickness ROI.

```
## [1] "Brain structure is Cerebral_peduncle_L"
##           Name         ep -2LL    df   AIC     diffLL diffdf p
## Model 1 : SibSaturated 42 5132.85 1942 1248.85 -      -      -
## Model 2 : eqMeSibs     36 5143.31 1948 1247.31 10.46  6      0.11
## Model 3 : eqMeZyg      32 5144.27 1952 1240.27 11.42  10     0.33
##
##           Name         ep -2LL    df   AIC     diffLL diffdf p
## Model 1 : SibSaturated 42 5132.85 1942 1248.85 -      -      -
## Model 2 : eqThSibs     36 5147.37 1948 1251.37 14.52  6      0.02
## Model 3 : eqThZyg      32 5151.24 1952 1247.24 18.39  10     0.05
```

```
##
## [1] "Brain structure is Fornix_cres_L"
##            Name          ep -2LL    df   AIC     diffLL diffdf p
## Model 1 : SibSaturated 42 5281.12 1942 1397.12 -      -      -
## Model 2 : eqMeSibs     36 5283.99 1948 1387.99 2.87   6      0.83
## Model 3 : eqMeZyg      32 5289.6  1952 1385.6  8.48   10     0.58
##
##            Name          ep -2LL    df   AIC     diffLL diffdf p
## Model 1 : SibSaturated 42 5281.12 1942 1397.12 -      -      -
## Model 2 : eqThSibs     36 5290.26 1948 1394.26 9.14   6      0.17
## Model 3 : eqThZyg      32 5295.62 1952 1391.62 14.5   10     0.15
##
## [1] "Brain structure is Fornix_cres_R"
##            Name          ep -2LL    df   AIC     diffLL diffdf p
## Model 1 : SibSaturated 42 5254.63 1942 1370.63 -      -      -
## Model 2 : eqMeSibs     36 5256.99 1948 1360.99 2.36   6      0.88
## Model 3 : eqMeZyg      32 5265.24 1952 1361.24 10.61  10     0.39
##
##            Name          ep -2LL    df   AIC     diffLL diffdf p
## Model 1 : SibSaturated 42 5254.63 1942 1370.63 -      -      -
## Model 2 : eqThSibs     36 5266.87 1948 1370.87 12.24  6      0.06
## Model 3 : eqThZyg      32 5276.1  1952 1372.1  21.47  10     0.02
##
## [1] "Brain structure is Cerebral_peduncle_R"
##            Name          ep -2LL    df   AIC     diffLL diffdf p
## Model 1 : SibSaturated 42 5177.25 1942 1293.25 -      -      -
## Model 2 : eqMeSibs     36 5185.87 1948 1289.87 8.62   6      0.2
## Model 3 : eqMeZyg      32 5187.51 1952 1283.51 10.26  10     0.42
##
##            Name          ep -2LL    df   AIC     diffLL diffdf p
## Model 1 : SibSaturated 42 5177.25 1942 1293.25 -      -      -
## Model 2 : eqThSibs     36 5186.85 1948 1290.85 9.6    6      0.14
## Model 3 : eqThZyg      32 5190.59 1952 1286.59 13.34  10     0.21
##
## [1] "Brain structure is Pontine_crossing_tract"
##            Name          ep -2LL    df   AIC     diffLL diffdf p
## Model 1 : SibSaturated 42 5260.08 1942 1376.08 -      -      -
## Model 2 : eqMeSibs     36 5263.04 1948 1367.04 2.96   6      0.81
## Model 3 : eqMeZyg      32 5266.68 1952 1362.68 6.59   10     0.76
##
##            Name          ep -2LL    df   AIC     diffLL diffdf p
## Model 1 : SibSaturated 42 5260.08 1942 1376.08 -      -      -
## Model 2 : eqThSibs     36 5273.59 1948 1377.59 13.51  6      0.04
## Model 3 : eqThZyg      32 5281.42 1952 1377.42 21.34  10     0.02
##
## [1] "Brain structure is Sagittal_stratum_L"
##            Name          ep -2LL    df   AIC     diffLL diffdf p
## Model 1 : SibSaturated 42 5247.62 1942 1363.62 -      -      -
## Model 2 : eqMeSibs     36 5255.13 1948 1359.13 7.51   6      0.28
## Model 3 : eqMeZyg      32 5256.83 1952 1352.83 9.21   10     0.51
##
##            Name          ep -2LL    df   AIC     diffLL diffdf p
## Model 1 : SibSaturated 42 5247.62 1942 1363.62 -      -      -
## Model 2 : eqThSibs     36 5256.11 1948 1360.11 8.49   6      0.2
```

```
## Model 3 : eqThZyg      32 5264.64 1952 1360.64 17.02  10     0.07
##
## [1] "Brain structure is Cingulum_hippocampus_R"
##           Name         ep -2LL    df   AIC     diffLL diffdf p
## Model 1 : SibSaturated 42 5283.08 1942 1399.08 -      -      -
## Model 2 : eqMeSibs     36 5290.44 1948 1394.44 7.36   6      0.29
## Model 3 : eqMeZyg      32 5292.45 1952 1388.45 9.37   10     0.5
##
##           Name         ep -2LL    df   AIC     diffLL diffdf p
## Model 1 : SibSaturated 42 5283.08 1942 1399.08 -      -      -
## Model 2 : eqThSibs     36 5290.96 1948 1394.96 7.88   6      0.25
## Model 3 : eqThZyg      32 5296.81 1952 1392.81 13.73  10     0.19
##
## [1] "Brain structure is Retrolenticular_part_internal_capsule_R"
##           Name         ep -2LL    df   AIC     diffLL diffdf p
## Model 1 : SibSaturated 42 5214.22 1942 1330.22 -      -      -
## Model 2 : eqMeSibs     36 5218.67 1948 1322.67 4.45   6      0.62
## Model 3 : eqMeZyg      32 5222.76 1952 1318.76 8.54   10     0.58
##
##           Name         ep -2LL    df   AIC     diffLL diffdf p
## Model 1 : SibSaturated 42 5214.22 1942 1330.22 -      -      -
## Model 2 : eqThSibs     36 5221.8  1948 1325.8  7.57   6      0.27
## Model 3 : eqThZyg      32 5228.25 1952 1324.25 14.03  10     0.17
##
## [1] "Brain structure is Cingulum_hippocampus_L"
##           Name         ep -2LL    df   AIC     diffLL diffdf p
## Model 1 : SibSaturated 42 5283.45 1942 1399.45 -      -      -
## Model 2 : eqMeSibs     36 5287.51 1948 1391.51 4.07   6      0.67
## Model 3 : eqMeZyg      32 5289.79 1952 1385.79 6.34   10     0.79
##
##           Name         ep -2LL    df   AIC     diffLL diffdf p
## Model 1 : SibSaturated 42 5283.45 1942 1399.45 -      -      -
## Model 2 : eqThSibs     36 5295.39 1948 1399.39 11.95  6      0.06
## Model 3 : eqThZyg      32 5300.1  1952 1396.1  16.65  10     0.08
##
## [1] "Brain structure is Corticospinal_tract_L"
##           Name         ep -2LL    df   AIC     diffLL diffdf p
## Model 1 : SibSaturated 42 5278.53 1942 1394.53 -      -      -
## Model 2 : eqMeSibs     36 5284.37 1948 1388.37 5.84   6      0.44
## Model 3 : eqMeZyg      32 5295.53 1952 1391.53 17.01  10     0.07
##
##           Name         ep -2LL    df   AIC     diffLL diffdf p
## Model 1 : SibSaturated 42 5278.53 1942 1394.53 -      -      -
## Model 2 : eqThSibs     36 5290.37 1948 1394.37 11.84  6      0.07
## Model 3 : eqThZyg      32 5295.1  1952 1391.1  16.57  10     0.08
##
## [1] "Brain structure is Superior_frontooccipital_fasciculus_R"
##           Name         ep -2LL    df   AIC     diffLL diffdf p
## Model 1 : SibSaturated 42 5304.7  1942 1420.7  -      -      -
## Model 2 : eqMeSibs     36 5307.73 1948 1411.73 3.02   6      0.81
## Model 3 : eqMeZyg      32 5322.15 1952 1418.15 17.45  10     0.07
##
##           Name         ep -2LL    df   AIC     diffLL diffdf p
## Model 1 : SibSaturated 42 5304.7  1942 1420.7  -      -      -
```

```
## Model 2 : eqThSibs     36 5311.99 1948 1415.99 7.28   6      0.3
## Model 3 : eqThZyg      32 5319.51 1952 1415.51 14.8   10     0.14
##
## [1] "Brain structure is Inferior_cerebellar_peduncle_R"
##           Name         ep -2LL    df   AIC     diffLL diffdf p
## Model 1 : SibSaturated 42 5259.36 1942 1375.36 -      -      -
## Model 2 : eqMeSibs     36 5263.02 1948 1367.02 3.66   6      0.72
## Model 3 : eqMeZyg      32 5278.17 1952 1374.17 18.81  10     0.04
##
##           Name         ep -2LL    df   AIC     diffLL diffdf p
## Model 1 : SibSaturated 42 5259.36 1942 1375.36 -      -      -
## Model 2 : eqThSibs     36 5277.7  1948 1381.7  18.34  6      0.01
## Model 3 : eqThZyg      32 5284.86 1952 1380.86 25.5   10     0
##
## [1] "Brain structure is FS_R_Superiorparietal_Thck"
##           Name         ep -2LL    df   AIC     diffLL diffdf p
## Model 1 : SibSaturated 42 5614.71 2074 1466.71 -      -      -
## Model 2 : eqMeSibs     36 5616.51 2080 1456.51 1.8    6      0.94
## Model 3 : eqMeZyg      32 5619.82 2084 1451.82 5.11   10     0.88
##
##           Name         ep -2LL    df   AIC     diffLL diffdf p
## Model 1 : SibSaturated 42 5614.71 2074 1466.71 -      -      -
## Model 2 : eqThSibs     36 5621.1  2080 1461.1  6.39   6      0.38
## Model 3 : eqThZyg      32 5625.6  2084 1457.6  10.89  10     0.37
```

The next set of model comparisons compares the fully saturated model to the ACTwE model, and then the ACTwE model to the AE model.

A separate set of output is provided for each FA and cortical thickness ROI.

```
## [1] "Brain structure is Cerebral_peduncle_L"
##           Name         ep -2LL    df   AIC     diffLL diffdf p
## Model 1 : SibSaturated 42 5132.85 1942 1248.85 -      -      -
## Model 2 : ACTwE        14 5180.28 1970 1240.28 47.43  28     0.01
##
##           Name  ep -2LL    df   AIC     diffLL diffdf p
## Model 1 : ACTwE 14 5180.28 1970 1240.28 -      -      -
## Model 2 : AE    8  5190.02 1976 1238.02 9.74   6      0.14
##
## [1] "Brain structure is Fornix_cres_L"
##           Name         ep -2LL    df   AIC     diffLL diffdf p
## Model 1 : SibSaturated 42 5281.12 1942 1397.12 -      -      -
## Model 2 : ACTwE        14 5313.29 1970 1373.29 32.17  28     0.27
##
##           Name  ep -2LL    df   AIC     diffLL diffdf p
## Model 1 : ACTwE 14 5313.29 1970 1373.29 -      -      -
## Model 2 : AE    8  5316.38 1976 1364.38 3.09   6      0.8
##
## [1] "Brain structure is Fornix_cres_R"
##           Name         ep -2LL    df   AIC     diffLL diffdf p
## Model 1 : SibSaturated 42 5254.63 1942 1370.63 -      -      -
## Model 2 : ACTwE        14 5293.54 1970 1353.54 38.91  28     0.08
##
##           Name  ep -2LL    df   AIC     diffLL diffdf p
```

```
## Model 1 : ACTwE 14 5293.54 1970 1353.54 -      -      -
## Model 2 : AE    8  5297.28 1976 1345.28 3.74   6      0.71
##
## [1] "Brain structure is Cerebral_peduncle_R"
##           Name         ep -2LL    df   AIC     diffLL diffdf p
## Model 1 : SibSaturated 42 5177.25 1942 1293.25 -      -      -
## Model 2 : ACTwE        14 5212.12 1970 1272.12 34.88  28     0.17
##
##           Name  ep -2LL    df   AIC     diffLL diffdf p
## Model 1 : ACTwE 14 5212.12 1970 1272.12 -      -      -
## Model 2 : AE    8  5221.73 1976 1269.73 9.61   6      0.14
##
## [1] "Brain structure is Pontine_crossing_tract"
##           Name         ep -2LL    df   AIC     diffLL diffdf p
## Model 1 : SibSaturated 42 5260.08 1942 1376.08 -      -      -
## Model 2 : ACTwE        14 5305.93 1970 1365.93 45.85  28     0.02
##
##           Name  ep -2LL    df   AIC     diffLL diffdf p
## Model 1 : ACTwE 14 5305.93 1970 1365.93 -      -      -
## Model 2 : AE    8  5310.3  1976 1358.3  4.38   6      0.63
##
## [1] "Brain structure is Sagittal_stratum_L"
##           Name         ep -2LL    df   AIC     diffLL diffdf p
## Model 1 : SibSaturated 42 5247.62 1942 1363.62 -      -      -
## Model 2 : ACTwE        14 5287.84 1970 1347.84 40.22  28     0.06
##
##           Name  ep -2LL    df   AIC     diffLL diffdf p
## Model 1 : ACTwE 14 5287.84 1970 1347.84 -      -      -
## Model 2 : AE    8  5291.15 1976 1339.15 3.31   6      0.77
##
## [1] "Brain structure is Cingulum_hippocampus_R"
##           Name         ep -2LL    df   AIC     diffLL diffdf p
## Model 1 : SibSaturated 42 5283.08 1942 1399.08 -      -      -
## Model 2 : ACTwE        14 5313.9  1970 1373.9  30.82  28     0.33
##
##           Name  ep -2LL    df   AIC     diffLL diffdf p
## Model 1 : ACTwE 14 5313.9  1970 1373.9  -      -      -
## Model 2 : AE    8  5317.92 1976 1365.92 4.03   6      0.67
##
## [1] "Brain structure is Retrolenticular_part_internal_capsule_R"
##           Name         ep -2LL    df   AIC     diffLL diffdf p
## Model 1 : SibSaturated 42 5214.22 1942 1330.22 -      -      -
## Model 2 : ACTwE        14 5249.92 1970 1309.92 35.7   28     0.15
##
##           Name  ep -2LL    df   AIC     diffLL diffdf p
## Model 1 : ACTwE 14 5249.92 1970 1309.92 -      -      -
## Model 2 : AE    8  5254.55 1976 1302.55 4.62   6      0.59
##
## [1] "Brain structure is Cingulum_hippocampus_L"
##           Name         ep -2LL    df   AIC     diffLL diffdf p
## Model 1 : SibSaturated 42 5283.45 1942 1399.45 -      -      -
## Model 2 : ACTwE        14 5321    1970 1381    37.55  28     0.11
##
##           Name  ep -2LL    df   AIC     diffLL diffdf p
```

```
## Model 1 : ACTwE 14 5321    1970 1381    -      -      -
## Model 2 : AE    8  5326.19 1976 1374.19 5.19   6      0.52
##
## [1] "Brain structure is Corticospinal_tract_L"
##           Name         ep -2LL    df   AIC     diffLL diffdf p
## Model 1 : SibSaturated 42 5278.53 1942 1394.53 -      -      -
## Model 2 : ACTwE        14 5324.93 1970 1384.93 46.4   28     0.02
##
##           Name  ep -2LL    df   AIC     diffLL diffdf p
## Model 1 : ACTwE 14 5324.93 1970 1384.93 -      -      -
## Model 2 : AE    8  5330.16 1976 1378.16 5.23   6      0.51
##
## [1] "Brain structure is Superior_frontooccipital_fasciculus_R"
##           Name         ep -2LL    df   AIC     diffLL diffdf p
## Model 1 : SibSaturated 42 5304.7  1942 1420.7  -      -      -
## Model 2 : ACTwE        14 5344.31 1970 1404.31 39.6   28     0.07
##
##           Name  ep -2LL    df   AIC     diffLL diffdf p
## Model 1 : ACTwE 14 5344.31 1970 1404.31 -      -      -
## Model 2 : AE    8  5351.73 1976 1399.73 7.42   6      0.28
##
## [1] "Brain structure is Inferior_cerebellar_peduncle_R"
##           Name         ep -2LL    df   AIC     diffLL diffdf p
## Model 1 : SibSaturated 42 5259.36 1942 1375.36 -      -      -
## Model 2 : ACTwE        14 5312.31 1970 1372.31 52.95  28     0
##
##           Name  ep -2LL    df   AIC     diffLL diffdf p
## Model 1 : ACTwE 14 5312.31 1970 1372.31 -      -      -
## Model 2 : AE    8  5317.52 1976 1365.52 5.21   6      0.52
##
## [1] "Brain structure is FS_R_Superiorparietal_Thck"
##           Name         ep -2LL    df   AIC     diffLL diffdf p
## Model 1 : SibSaturated 42 5614.71 2074 1466.71 -      -      -
## Model 2 : ACTwE        14 5647.15 2102 1443.15 32.43  28     0.26
##
##           Name  ep -2LL    df   AIC     diffLL diffdf p
## Model 1 : ACTwE 14 5647.15 2102 1443.15 -      -      -
## Model 2 : AE    8  5648.01 2108 1432.01 0.86   6      0.99
```

Section 4: White Matter Fractional Anisotropy

Standardized estimates and 95% confidence intervals for each of the paths in the bivariate cholesky decomposition model are provided for each of the 12 white matter ROIs in **Figure S1**.

These standardized estimates were then converted into variances and used to visualize how much of variation in FA values and BMI could be attributed to each of the four sources. For example, in the ACTwE model, white matter FA variance is defined as below:

$$Var_{FA} = Var_{FA_A} + Var_{FA_C} + Var_{FA_{Tw}} + Var_{FA_E}$$

For white matter FA, each of the variance components is the square of the respective standardized loading For example,

$$Var_{FA_A} = a_{11}^2$$

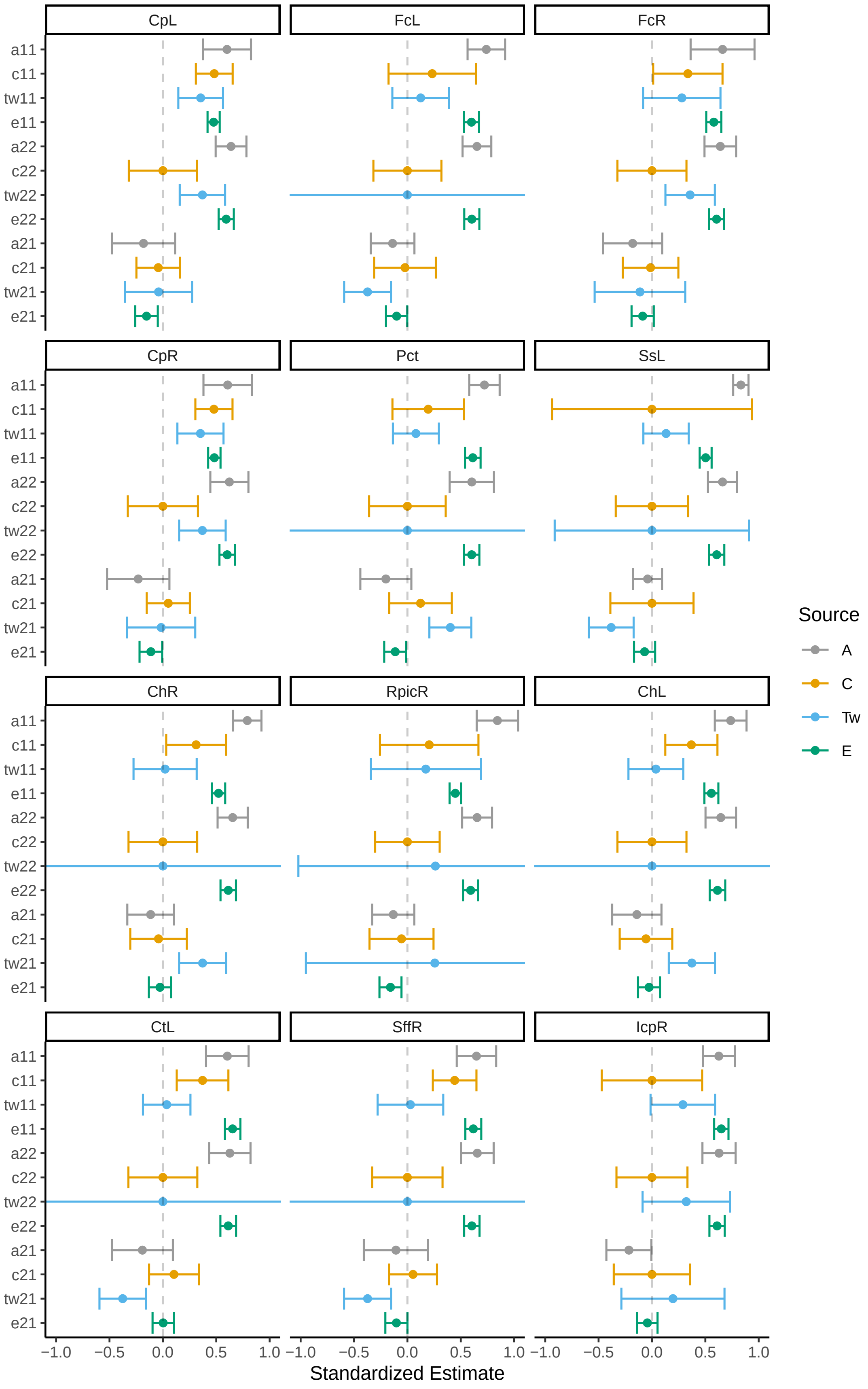

Figure S1: Behavioral genetic path estimates for BMI and white matter FA

For BMI, each of the variance components is the sum of the respective squared cross-loading and the squared endurance-specific standardized loading. For example,

$$Var_{BMI_A} = a_{21}^2 + a_{22}^2$$

The estimates from the ACTwE model can also be used to partition the phenotypic correlation into A, C, Tw and E components. For example, the portion of the correlation attributed to heritability can be estimated by first estimating the covariance:

$$Cov_A = a_{11} \times a_{21}$$

And then coverting that value into a correlation.

$$Cor_A = \frac{Cov_A}{(Var_{FA_A} + Var_{FA_C} + Var_{FA_{Tw}} + Var_{FA_E})^{\frac{1}{2}} \times (Var_{BMI_A} + Var_{BMI_C} + Var_{BMI_{Tw}} + Var_{BMI_E})^{\frac{1}{2}}}$$

Proportions of the variances and of correlations are plotted in **Figure S2**.

Section 5: Gray Matter Structure

All of the above behavioral genetic analyses were repeated on the right superior pariatal thickness, with the results summarized in **Figure S3**.

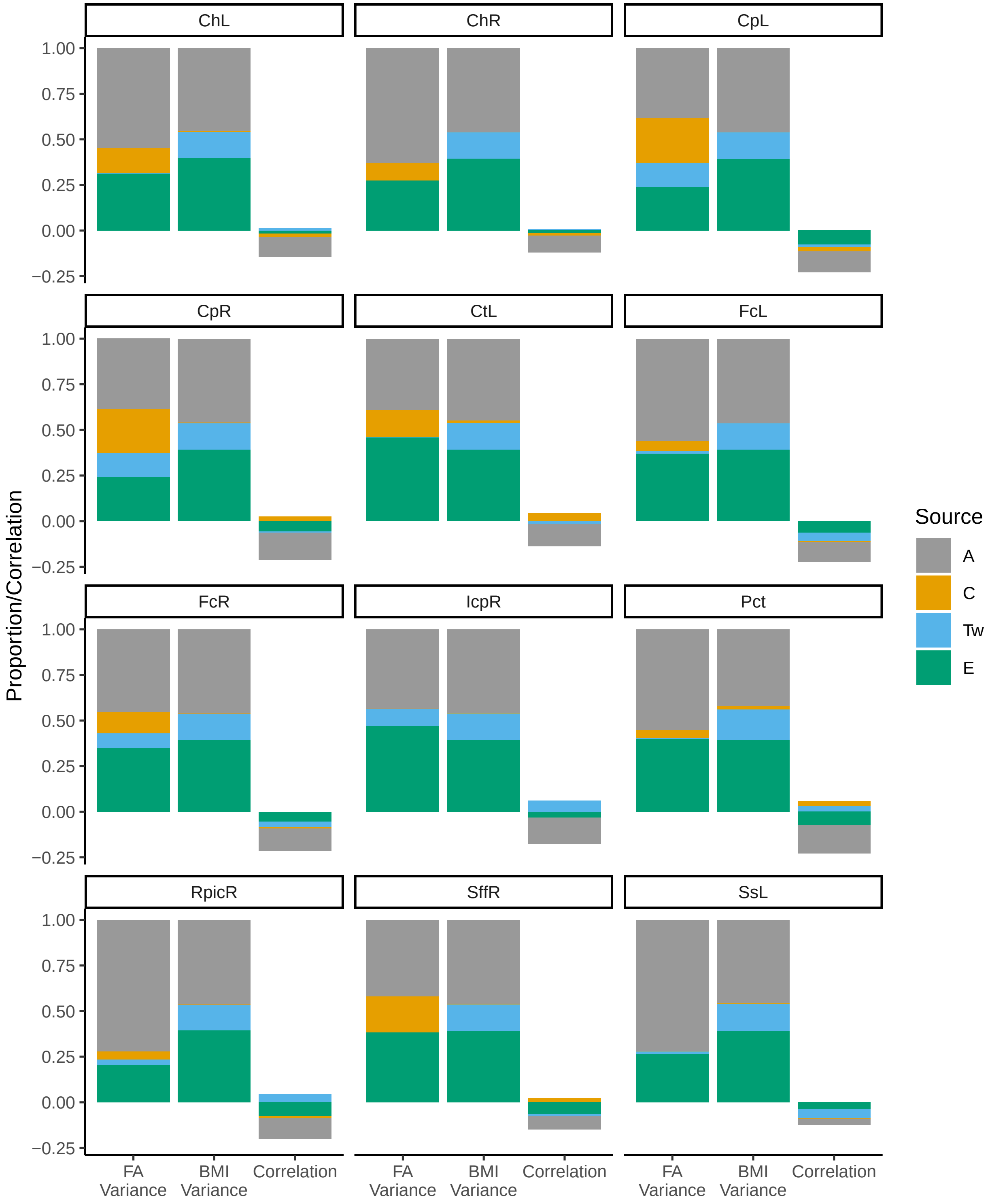

Figure S2: Variance components for BMI and white matter FA

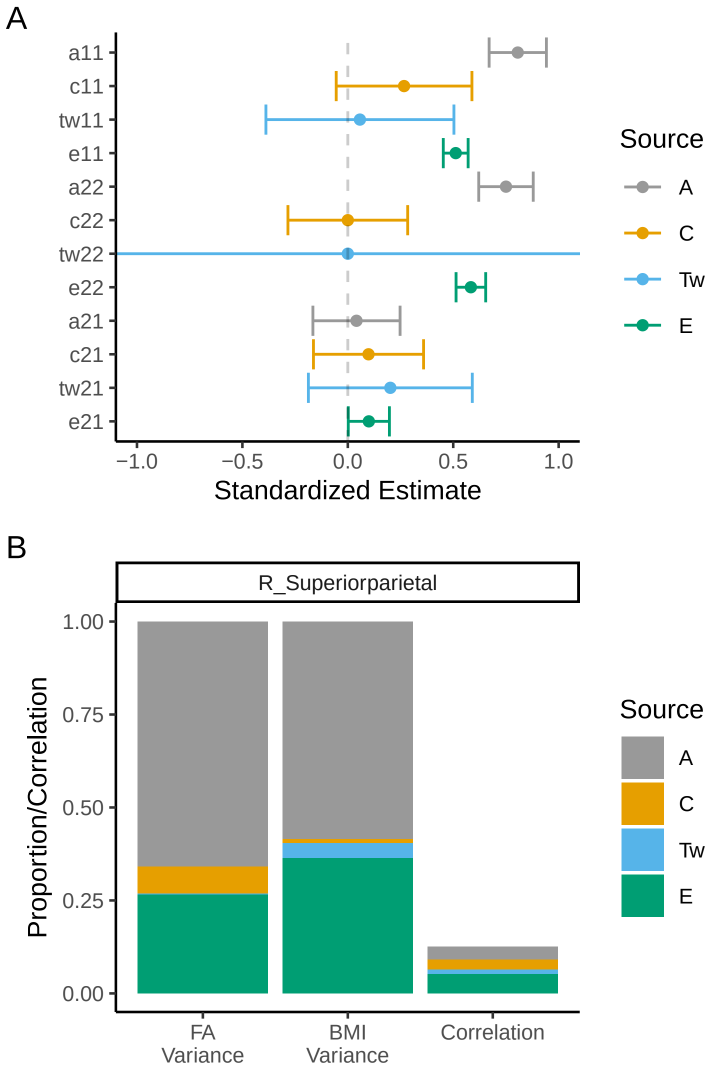

Figure S3: Summary of behavioral genetic analyses of BMI and right superior parietal thickness
